# Malignant Tracheal Stenosis: A Narrative Review of Current Diagnostic and Therapeutic Approaches

**DOI:** 10.3390/jcm15197638

**Published:** 2026-10-02

**Authors:** Cornel Florentin Savu, Amir-Andrei Sabha, Mihaela-Beatrice Tivadar, Silviu Gabriel Vlasceanu, Mihnea Orghidan, Andrei-Cristian Bobocea

**Affiliations:** 1 Department of Thoracic Surgery, “Marius Nasta” National Institute of Pneumology, 050150 Bucharest, Romania; drsavu25@yahoo.com (C.F.S.); tivadarbeatrice@gmail.com (M.-B.T.); mihnea.org@gmail.com (M.O.); andrei.bobocea@gmail.com (A.-C.B.); 2 Thoracic Surgery I, University of Medicine and Pharmacy “Carol Davila”, 020021 Bucharest, Romania

**Keywords:** malignant tracheal stenosis, adenoid cystic carcinoma, squamous cell carcinoma, tracheal resection, interventional bronchoscopy, tracheal stent, thoracic surgery, palliative care

## Abstract

**Background:** Malignant tracheal stenosis (MTS) is a rare, life-threatening condition caused by primary tracheal tumor, mainly adenoid cystic carcinoma (ACC) and squamous cell carcinoma (SCC), and more rarely other primary neoplasms such as lymphomas, or local extension/metastasis from adjacent malignancies (thyroid, esophageal, lung, or mediastinal cancers). Accounting for <0.1% of respiratory tumors, MTS presents with progressive respiratory failure, requiring urgent multidisciplinary care involving thoracic surgery, interventional pulmonology, oncology, and intensive care. Diagnosis relies on clinical evaluation, advanced imaging and endoscopy. Bronchoscopy (BC) remains the best investigation for direct visualization and biopsy or performing therapeutic maneuvers. Prognosis depends on histology, local extent, and achieving R0 resection. Tracheal resection with primary anastomosis remains the curative standard for resectable cases. Interventional procedures (debulking, laser, cryotherapy, stenting) are essential for palliative management and acute stabilization. **Methods:** A structured (non-systematic) search of PubMed/MEDLINE, Embase, Scopus, and Web of Science (1965–2025) was performed using MeSH/Emtree terms and free-text keywords. Studies were included if they evaluated adult patients with malignant central airway stenosis, primary tracheal tumors, or secondary invasion undergoing surgical, bronchoscopic, or multimodal interventions, including ECMO support. **Results:** Malignant tracheal stenosis (MTS)—predominantly adenoid cystic and squamous cell carcinomas—imposes a critical clinical burden despite its low incidence. Complete surgical resection with negative margins remains the curative treatment for resectable disease. In unresectable cases, multimodal palliative interventions (stenting, laser/electrocautery ablation, and mechanical debulking) provide rapid airway restoration and quality-of-life improvements, whereas ECMO is an established rescue or adjunctive support strategy in selected critical airway procedures performed in experienced centers. Three-dimensional bioprinting and tissue-engineered reconstruction remain experimental or early clinical, and bioresorbable stents and artificial intelligence (AI)-based tools remain investigational; none is currently part of routine MTS management. **Conclusions:** R0 resection for appropriately selected resectable disease and interventional airway management for unresectable symptomatic disease are the best-supported strategies, although the evidence derives mainly from retrospective series from specialized centers. Emerging technologies require prospective validation before clinical adoption. Management should be individualized, multidisciplinary and centralized in experienced airway centers.

## 1. Introduction

Malignant tracheal stenosis (MTS) is a particular form of central airway obstruction characterized by the narrowing of the trachea as a result of either tumor invasion or extrinsic compression. As opposed to benign obstructions, which are usually associated with iatrogenic tracheal trauma or inflammatory disease, MTS appears due to primary tracheal tumors or secondary to adjacent tumors and thus has a less favorable prognosis [1,2].

The anatomical features of the trachea and its proximity to vital mediastinal structures account for the rapid decline in airway patency and the high complexity of surgical intervention in advanced cases [2,3,4].

Malignant tracheal tumors are rare, representing under 0.1% of respiratory neoplasms [5,6,7,8]. Nevertheless, their clinical impact outweighs their low incidence due to the high risk of acute respiratory failure and mortality without prompt intervention [1,2]. Advances in modern imaging and prolonged survival in cancer patients over recent decades have enabled more frequent, early-stage diagnosis of these lesions [9].

The foundations of modern tracheal surgery were established in the mid-to-late 20th century with the introduction of segmental resection and primary anastomosis, demonstrating that anatomical continuity and respiratory function could be restored [3,10]. Subsequent advances in anesthesia, ventilation strategies, suture materials, and tracheal release techniques have improved the safety of tracheal resection and reconstruction and reduced the risk of perioperative and anastomotic complications [4,11,12]. Simultaneously, progress in CT and video BC allowed for more accurate staging and refined patient selection for curative intervention [13,14,15].

Contemporary studies confirm that complete resection with negative margins is the primary determinant of long-term survival in malignant tracheal tumors [5,16,17]. Series published by Grillo et al. [3,18] and Gaissert et al. [7] demonstrate that, in experienced centres, tracheal surgery yields low mortality and favorable functional outcomes [16,19]. Concurrently, advances in interventional BC, including silicone and metallic tracheal stenting, have transformed the management of advanced or unresectable diseases, offering rapid airway restoration and symptomatic relief [20,21,22,23,24].

Extracorporeal membrane oxygenation (ECMO) has become an established rescue or adjunctive support strategy for selected complex airway procedures in experienced centers, whereas patient-specific stents and tissue-engineered reconstruction remain emerging approaches supported mainly by case series and experimental data [25,26,27]. Thus, managing MTS demands an individualized, multidisciplinary approach incorporating thoracic surgery, interventional pulmonology, medical and radiation oncology, anesthesiology, and critical care medicine [28,29,30].

## 2. Materials and Methods

### 2.1. Study Design and Methodological Rationale

This study was designed as a narrative literature review, analyzing and synthetizing the management of MTS from the perspectives of thoracic surgery, interventional BC, and oncology. Although a structured search of several databases was performed to limit selection bias, the review does not follow systematic review methodology (e.g., PRISMA); no formal risk-of-bias assessment or quantitative synthesis was undertaken, and the review does not claim systematic comprehensiveness. Case reports were included only for rare entities or techniques for which larger series are not available.

### 2.2. Search Strategy

A search was conducted between January 2024 and June 2025 across PubMed/MEDLINE, Embase, Scopus, and Web of Science. The strategy combined controlled vocabulary (MeSH/Emtree) and free-text keywords using Boolean operators to identify relevant studies across thoracic surgery, interventional BC, oncology, radiology, and critical care. Publications from 1965 to 2025 were analyzed to encompass both historical foundations and emerging therapeutic strategies. A representative Boolean search string applied in PubMed/MEDLINE was structured as follows:

(“Tracheal Neoplasms”[Mesh] OR “Airway Obstruction”[Mesh] OR “malignant tracheal stenosis”[TiAb] OR “tracheal tumor*”[TiAb] OR “tracheal cancer*”[TiAb] OR “central airway obstruction”[TiAb]) AND (“Trachea/surgery”[Mesh] OR “Stents”[Mesh] OR “Bronchoscopy”[Mesh] OR “tracheal resection”[TiAb] OR “interventional bronchoscopy”[TiAb] OR “airway stent*”[TiAb] OR “ECMO”[TiAb] OR “multimodal”[TiAb])

Equivalent search strings adapted for Embase, Scopus, and Web of Science syntax were utilized. Language filters were restricted to English-language peer-reviewed publications.

### 2.3. Inclusion and Exclusion Criteria

Studies included if they evaluated adult patients with malignant central airway stenosis/obstruction, primary tracheal tumors, or secondary invasion; reported outcomes on tracheal resection, reconstruction, interventional BC, stenting, or multimodal techniques; or evaluated ECMO or extracorporeal life support (ECLS) and emerging technologies. Retrospective cohorts, observational studies, multicenter series, key case reports, clinical guidelines, and narrative reviews were included. Studies lacking direct clinical relevance, duplicates, and papers with insufficient diagnostic or therapeutic data were excluded.

### 2.4. Selection Process and Final Corpus

Database queries were screened by title, abstract, and full text to identify relevant evidence. Following duplicate removal and eligibility screening, a final corpus of literature was selected for narrative synthesis, comprising original clinical studies, comprehensive review articles, and foundational references.

### 2.5. Analyzed Variables and Methodological Limitations

For each study, data were extracted regarding etiology, obstruction mechanism, therapeutic techniques, complications, airway patency, re-interventions, and overall survival, focusing on clinical relevance for specialized airway centers.

Primary limitations stem from the retrospective and heterogeneous nature of available literature, a lack of standardized evaluation criteria, and technological changes over time. Nevertheless, synthesizing an extensive corpus provides an up-to-date, clinically meaningful overview of contemporary MTS management.

Because the available literature combines historical surgical series, retrospective cohorts, registry analyses, case reports, experimental studies and reviews, these sources are not treated as equivalent levels of evidence. Throughout the text, the design of key studies is explicitly signposted (e.g., single-center retrospective series, population-based registry, case report, preclinical model), and it is stated when evidence derives from mixed central airway obstruction (CAO) cohorts, from benign stenoses or from non-tracheal airway sites rather than from patients with primary or secondary MTS; such data should be regarded as indirect evidence.

Textual draft segments were processed through Gemini (version 1.5/2.0, Google LLC, Mountain View, CA, USA) for grammar checking, spelling correction, and style refinement. The suggested edits were manually reviewed and verified by the authors to preserve technical accuracy and original intent.

## 3. Results

### 3.1. Epidemiology of Malignant Tracheal Stenosis

Primary tracheal tumors have an estimated annual incidence of 0.1–0.2 per 100,000 population, accounting for <0.1% of all respiratory malignancies [5,6,7,31,32,33,34,35]. Adenoid cystic carcinoma (ACC) and SCC predominate histologically, followed by mucoepidermoid carcinomas, neuroendocrine tumors, and rare sarcomas [6,8,36,37,38,39,40]. Secondary MTS is more common in clinical practice, arising via direct invasion or extrinsic compression from esophageal, thyroid, bronchopulmonary, or mediastinal neoplasms, as well as metastatic lymphadenopathy and mediastinal lymphomas [6,24,39,40,41]. True incidence remains underestimated due to frequent late-stage presentation [5,6].

- Sex and Age Distribution: a slight male predominance exists, predominantly in SCC cases linked to tobacco smoke and inhalational carcinogen exposure [6]. Conversely, ACC exhibits equal sex distribution and affects younger cohorts (aged 40–60 years) [5]. Overall mean age at diagnosis spans 52–60 years depending on histology [16,42].
- Geographic Distribution: primary tracheal tumor incidence is globally rather uniform. Secondary stenoses linked to esophageal carcinoma prevail in Asian populations, whereas ACC- and SCC-driven stenoses predominate across European and North American specialized thoracic centres [31,42,43]. While the baseline population incidence of primary tracheal malignancies is relatively uniform globally, reported geographic variations in secondary MTS and histological proportions should be interpreted cautiously. These variations are heavily influenced by tertiary referral center selection bias, local institutional specialization, and differences in registry capture methodologies rather than true biological disparities.

### 3.2. Etiology of Malignant Tracheal Stenosis

MTS stems from two primary etiologies: primary tracheal tumors and secondary stenoses resulting from local invasion or distant metastasis. Lesion growth, histological subtype, and locoregional invasiveness dictate stenosis progression, while prior cervicothoracic radiotherapy or oncological interventions can further induce fibrous remodeling and structural tracheal compromise [6,33,44,45].

#### 3.2.1. Primary Malignant Tracheal Stenosis

Primary tracheal tumors constitute 60–70% of surgical series in specialized centres [46]. Histologically, two types predominate:

- ACC ~40% of cases, characterized by indolent growth and extensive submucosal longitudinal spread [43,46,47].
- SCC ~20–30% of cases, strongly linked to tobacco use and higher biological aggressiveness [46,47,48].

Other rare primary tracheal tumors include mucoepidermoid carcinomas, neuroendocrine tumors, tracheal sarcomas, and primary lymphomas [29,48].

Primary tracheal lymphomas, most commonly extranodal marginal zone lymphomas of mucosa-associated lymphoid tissue (MALT), are exceptionally rare and differ fundamentally from epithelial carcinomas in biology, natural history and treatment: they usually follow an indolent course and are responsive to radiotherapy and anti-CD20-based systemic therapy. Because they often present as smooth submucosal or circumferential wall thickening, they may mimic cicatricial or post-intubation stenosis, chronic tracheitis, granulation tissue, benign endotracheal lesions or nonspecific inflammatory disease, and superficial endoscopic biopsies may be non-diagnostic. Topolnitskiy et al. described primary tracheal MALT lymphoma in an 81-year-old patient in whom preoperative endoscopic biopsy was non-diagnostic and the definitive diagnosis was established only after circumferential tracheal resection with histopathological and immunohistochemical examination [49]. Secondary tracheal involvement by systemic or mediastinal lymphomas, in contrast, usually causes extrinsic compression and is primarily an indication for systemic therapy.

#### 3.2.2. Secondary Malignant Tracheal Stenosis

Secondary lesions most frequently arise from direct invasion by esophageal, thyroid, or bronchopulmonary carcinomas [6,50,51,52,53]. Extrinsic compression by metastatic mediastinal lymphadenopathy can similarly precipitate severe, rapidly progressive airway obstruction [1,54]. Rarely, hematogenous metastases cause circumferential wall infiltration and acute respiratory distress, as described in renal cell, colorectal, breast, bladder and malignant melanoma cancers [6,55].

### 3.3. Classification of MTS

MTS can be classified based on anatomical extent (Table 1), degree of lumen loss, and underlying mechanism. Lacking a standardized TNM staging system for primary tracheal tumors due to rarity and histologic heterogeneity, clinical practice relies on anatomo-functional, morpho-functional, and pathophysiological classifications [33,39,56,57].

#### 3.3.1. Anatomo-Functional Classification

The current proposed classification systems aim to structure the diagnostic and therapeutic approach to MTS based on anatomical location, functional impact, and morphological characteristics.

#### 3.3.2. Morpho-Functional Classification

Popularized by Freitag [56] and Ernst [2] this scheme categorizes MTS by the structural relationship between tumor and airway wall:

- Intraluminal: Exophytic intraluminal tumor growth with preserved wall structure (typical of SCC or carcinoids).
- Intramural: Infiltration of the tracheal wall and cartilage leading to rigidity (typical of ACC).
- Extrinsic: External airway compression without mucosal invasion (e.g., invasive thyroid tumors, mediastinal lymphadenopathy).
- Mixed: Combined transmural wall infiltration and extrinsic compression.

#### 3.3.3. Integrated Clinicopathological Framework

Because primary epithelial tumors, rare primary neoplasms such as lymphomas, and secondary tracheal involvement differ in biology, prognosis, resectability and treatment goals, histological category and obstruction pattern should be considered together when defining the respective roles of surgery, bronchoscopic intervention, stenting and oncological therapy (Table 2). Primary epithelial tumors are the principal candidates for curative resection; primary lymphomas are managed mainly with non-surgical oncological therapy; and secondary involvement is usually approached with palliative bronchoscopic and primary tumor-directed oncological treatment, with resection reserved for selected cases such as differentiated thyroid carcinoma with limited airway invasion [6,44,60,61]. Superimposed on histology, the pattern of obstruction (endoluminal, intramural, extrinsic or mixed) determines which bronchoscopic modality and stent type are appropriate [2,56].

#### 3.3.4. Pathophysiological Classification & Severity Grading

Clinical urgency and therapeutic routing are stratified by luminal reduction according to the established Myer–Cotton grading system [63]:

- Grade I (Mild): <50% reduction; minimal exertional symptoms.
- Grade II (Moderate): 51–70% reduction; dyspnea on mild exertion.
- Grade III (Severe): 71–99% reduction; rest dyspnea, stridor.
- Grade IV (Critical): 100% reduction; imminent asphyxiation risk (medical emergency).

The Myer–Cotton system was originally proposed and validated for subglottic stenosis on the basis of endotracheal tube sizing [63]; its application to MTS is by analogy and has not been formally validated in this population. Moreover, the relationship between percentage stenosis, absolute residual lumen diameter, symptom severity and functional impairment is not linear, as symptoms also depend on stenosis length, dynamic components, baseline airway caliber, secretions and cardiopulmonary reserve. Grading should therefore be interpreted together with the absolute lumen diameter and the clinical status [2,56].

### 3.4. Diagnosis of Malignant Tracheal Stenosis

The diagnosis of MTS represents a complex undertaking that requires an integrated approach based on correlating clinical, imaging, and endoscopic data. Given the rarity and insidious nature of these lesions, early recognition is frequently delayed; clinical manifestations are often non-specific and misdiagnosed as far more prevalent respiratory diseases, such as bronchial asthma, chronic obstructive pulmonary disease, or bilateral vocal cord paralysis [1,7,8,39,40,48,64,65].

A complete diagnostic evaluation aims to:

- Determine stenosis etiology
- Assess the degree of luminal narrowing and exact location of the obstruction
- Evaluate tumor extension and anatomical relationships with adjacent structures
- Establish lesion resectability and available therapeutic options (curative vs. palliative).

#### 3.4.1. Clinical Manifestations

Clinical manifestations result directly from mechanical airway obstruction and span a wide spectrum of severity, ranging from minimal respiratory discomfort to life-threatening acute respiratory failure [1,2,35,66,67]. Progressive dyspnea is the cardinal symptom, its intensity correlates closely with the degree of tracheal lumen narrowing:

- Early Stages (Grades I–II): Exertional dyspnea predominates, corresponding to mild-to-moderate stenosis.
- Advanced Stages (Grade III): Rest dyspnea develops, frequently accompanied by inspiratory stridor, characteristic of severe stenosis.
- Critical Cases (Grade IV): Marked airway obstruction causes life-threatening severe dyspnea, hypoxemia, intense anxiety, and signs of acute respiratory distress (Table 3).

#### 3.4.2. Pulmonary Function Testing

Spirometry with flow-volume loop analysis lacks diagnostic specificity but provides valuable insights into obstruction severity. Flattening of both inspiratory and expiratory loops typically indicates fixed central airway obstruction [46,54]. Pulmonary function testing is useful for longitudinal monitoring and post-stenting or surgical treatment evaluation [46,68].

#### 3.4.3. Diagnostic Imaging

Diagnostic imaging plays a central role in evaluating malignant tracheal stenosis, defining lesion location, extent, and obstructive mechanism [2,9,14,15].

- Chest Radiography: Standard radiographs have low sensitivity but may indirectly reveal tracheal air column narrowing, tracheal deviation, or mediastinal masses. Its utility is primarily preliminary [2,54].
- CT: Thoracic CT is the most useful imaging modality [2,69]. It allows precise assessment of: stenosis location and length, luminal narrowing degree, structural relationships with mediastinal anatomy, cartilaginous and peritracheal invasion, and associated lymphadenopathy. Furthermore, 3D reconstructions and virtual BC further aid surgical and endoscopic planning [25,38,70]. CT and BC have complementary rather than competing roles: CT provides anatomical mapping, assessment of extraluminal extension and surgical planning, whereas BC provides direct assessment of the airway lumen and tissue acquisition.
- Magnetic Resonance Imaging (MRI): MRI serves a complementary role, particularly useful for evaluating tumor extension into soft tissues, mediastinal vasculature, or the thyroid gland [71].
- Positron emission tomography (PET-CT): is employed for oncological staging and detecting distant metastases, assisting in resectability determination for borderline cases [15,72].

#### 3.4.4. Endoscopic Diagnostic Methods

Rigid and flexible BC represent the method of choice for confirming diagnosis of MTS, offering a direct morphological assessment, as well as the possibility to perform biopsies in order to obtain a histologic confirmation [13,38,73]. By direct visualization many details can be obtained about the localization of the stenosis, its extent longitudinally as well as circumferentially and the degree of obstruction (Figure 1), as well as the characteristics of the tumor itself [74,75,76]. Furthermore, it can evaluate the dynamic of the airway and the measurement of the distance from the MTS to the vocal cords and carina, which provides valuable information for planning future treatment. A major advantage of BC is that it offers the possibility to perform therapeutic interventions such as tumor debulking, dilation or stenting [73,77].

Rigid BC is preferred in severe stenoses due to the superior airway control and the possibility to ventilate, thus being indispensable in complex interventional procedures [75,78]. Flexible BC, however, is predominantly used as initial assessment method, in order to obtain biopsies and for postoperative monitoring [13,77]. For patients with critical stenosis, sedation must be done with extreme precaution, since there is a risk of airway collapse due to loss of glottic tonus. In these situations, procedures are done only in specialized centres, and sometimes even with extracorporeal circulatory support [11,79,80].

In practical terms, flexible BC—ideally performed in a setting where rigid BC is immediately available—is suitable for initial assessment and biopsy in patients with non-critical stenosis and a stable airway. Rigid BC under general anesthesia is preferred when the residual lumen is critical, when the lesion is highly vascular or friable, when therapeutic recanalization (core-out, dilation or stent placement) is anticipated in the same session, or when a flexible examination could precipitate complete obstruction. Extracorporeal support should be considered and planned in advance when conventional ventilation cannot be ensured, e.g., near-complete obstruction of the distal trachea or carina, anticipated loss of the airway during induction, or complex airway-off procedures; in such cases ECMO is best established before induction of anesthesia [11,30,79,81].

Advanced endoscopic techniques contribute to detailed lesion characterization and accurate disease staging. Autofluorescence endoscopy differentiates tumor tissue from normal mucosa by highlighting fluorescence alterations associated with malignant transformation [13,29]. Narrow Band Imaging (NBI) optimizes visualization of pathological vascular patterns and facilitates mapping of superficial mucosal extension [13].

Endobronchial ultrasound (EBUS) plays a key role in evaluating submucosal extension and identifying paratracheal lymphadenopathy, providing critical data for staging and surgical or oncological treatment planning [72,82,83].

#### 3.4.5. Histopathological and Immunohistochemical Diagnosis

Definitive diagnosis of MTS is based on histopathological examination of tissue obtained through endoscopic biopsy or of the surgically removed specimen. Tumor type, differentiation grade, perineural/vascular invasion, and surgical margin status are the evaluated parameters [2,39].

ACC is characterized by cribriform or tubular structures composed of basaloid cells, exhibiting submucosal longitudinal extension and perineural invasion responsible for late recurrences [32]. SCC consists of polygonal cells with nuclear atypia and variable keratinization, frequently showing deep mural invasion and regional lymph node metastasis [84]. Lastly, neuroendocrine tumors display an organoid architecture and variable mitotic index [85,86].

Immunohistochemistry (IHC) is essential for precise classification and determining tumor origin: ACC expresses CD117, cytokeratins, and myoepithelial markers; SCC express squamous differentiation markers (p40, p63). Secondary lesions are differentiated via panel-specific markers (e.g., TTF-1 for lung adenocarcinoma, PAX8 for thyroid origin, CD20/CD3 for lymphomas). Integrating histopathology with IHC is critical for prognostic estimation, assessing microscopic extension, and guiding surgical or adjuvant strategies [87]. Overall, these analyses provide essential insights into tumor biology, staging, and management.

#### 3.4.6. Differential Diagnosis

MTS must be distinguished from benign and tumor-like conditions that produce similar clinical and endoscopic findings, including post-intubation or post-tracheostomy stenosis, idiopathic subglottic stenosis, granulomatosis with polyangiitis, relapsing polychondritis, amyloidosis and benign tracheal tumors [1,2,38]. Rare tumor-like disorders may also mimic malignancy. Rosai–Dorfman disease, a non-Langerhans cell histiocytosis rather than a malignant neoplasm, can cause clinically significant subglottic and tracheal obstruction: Topolnitskiy et al. reported a 71-year-old patient with critical multifocal subglottic and tracheal obstruction, in whom the diagnosis relied on histiocytic infiltrates with emperipolesis and immunohistochemistry, and who was treated with laryngotracheal reconstruction, cryosurgery and subsequent chemotherapy [88]. Similarly, primary tracheal MALT lymphoma may mimic cicatricial stenosis or chronic inflammatory disease, and superficial biopsies may be non-diagnostic [49]. Atypical stenotic lesions, particularly those with smooth mucosa or submucosal/infiltrative growth, therefore require adequate deep tissue sampling and comprehensive histopathological and immunohistochemical evaluation before definitive treatment, and a non-diagnostic superficial biopsy should not be considered reassuring.

#### 3.4.7. Proposed Diagnostic Algorithm

Diagnosing MTS requires a systematic, phased approach to ensure early identification, complete characterization, and appropriate therapeutic selection (Figure 2). Slowly progressive dyspnea and stridor, refractory to bronchodilators raises the clinical suspicion of MTS. Standard radiography is usually the first investigation for screening and further orienting the diagnosis. The standard investigation is a chest CT which offers valuable information regarding the degree of stenosis, whether it is a primary tracheal tumor or extrinsic compression from tumors of surrounding organs, it allows 3D reconstructions, measuring the length and localization of the tracheal stenosed segment, the degree of obstruction and lastly the relation to adjacent structures [9,14,37].

BC offers the possibility to directly view the cause of MTS, to perform biopsies and, if needed, perform therapeutic maneuvers such as dilation or stenting [13,22]. As for any oncologic case, assessment of systemic involvement is needed and thus PET-CT and MRI may be used to see the extent of the disease and then the case must be reviewed by a specialized Tumor Board to determine the precise course of action for each specific patient [15,28].

### 3.5. Surgical Management

Surgical treatment is the main curative intent treatment modality for resectable primary tracheal malignancies and for certain selected cases of localized secondary tumors. The goal is R0 resection with clear margins and anatomical airway reconstruction [3,17,36]. Patient selection requires rigorous multidisciplinary evaluation based on tumor extent, lesion length, oncological status, and cardiorespiratory reserve [30,89].

#### 3.5.1. Indications

- Resectable primary tracheal tumors (ACC, SCC, carcinoid, mucoepidermoid) [18,47,60].
- Resection length (less than 5–6 cm or <50% of total tracheal length in adults) to enable a tension-free anastomosis; this is an indicative rather than a universal threshold (see below) [4].
- Localized malignant stenosis without extensive vital organ invasion [44,51].
- Preoperative imaging/BC confirming clear proximal/distal margins, with intraoperative frozen sections targeting an optimal oncological safety margin of >5 mm [16,17].
- Absence of distant metastases and acceptable anesthetic risk [30,31,60].

#### 3.5.2. Relative Indications & Selected Cases

- Limited en bloc resection for local invasion into the esophagus or thyroid gland [5,31,50,52,60,61].
- Local recurrence post-radiotherapy provided safe reconstruction is feasible [16].
- Indolent tumors (e.g., ACC) with microscopic positive margins when complete excision is technically unfeasible, as adjuvant therapy can control local disease [50].

#### 3.5.3. Contraindications

- Carinal involvement too extensive for safe reconstruction, or extensive invasion of the great vessels or pericardium [19,82].
- Distant metastases, severe cardiorespiratory impairment, or poor performance status (orienting care toward palliative interventions) [41,65].

Tracheal involvement > 6 cm is frequently considered beyond the limits of safe resection due to the high risk of anastomotic tension [16,59], but other aspects should be considered when establishing resectability (patient characteristics, tissue mobility, release maneuvers, prior radiation, institutional expertise). There have been reports of resections of up to 6.5 to 7 cm in rare highly selected cases [90].

Resection length should therefore be regarded as an important but individualized determinant of technical feasibility rather than a fixed criterion of resectability. Moreover, these thresholds, as well as the survival and recurrence data discussed below, derive largely from small retrospective series of epithelial tumors treated in highly specialized centers; they should be interpreted cautiously and should not be directly extrapolated to other settings or to tracheal lymphomas.

#### 3.5.4. Primary Versus Secondary Tracheal Involvement

Treatment indications differ between primary and secondary disease. For primary tracheal malignancies, resection with curative intent is the treatment of choice whenever complete resection is feasible, followed by adjuvant radiotherapy for positive margins or perineural invasion, whereas definitive radiotherapy is used for unresectable disease and systemic therapy has a limited, mainly palliative role [16,28,32]. For secondary involvement, resection is limited to selected cases, mainly differentiated thyroid carcinoma with limited tracheal invasion, in which en bloc resection offers durable local control [51,60,61]; airway invasion by esophageal or lung cancer and metastatic involvement are generally treated with primary tumor-directed systemic and radiation therapy, combined with bronchoscopic recanalization and stenting for symptomatic obstruction [44,52,53].

#### 3.5.5. Special Considerations: Primary Tracheal Lymphoma

Tracheal lymphomas represent a distinct scenario in which the surgical principles derived from epithelial tumors should not be applied directly. Given the typically indolent course of MALT lymphoma and its responsiveness to radiotherapy and systemic therapy, surgical resection is not a universal standard. It may be appropriate in selected patients with critical airway obstruction requiring urgent restoration, when less invasive biopsies are non-diagnostic, or for localized resectable disease without systemic involvement [49]. Published reports describe local resection, bronchoscopic therapy, radiotherapy, rituximab-containing therapy and immunochemotherapy, and no single established standard exists [62]. Management should therefore be individualized through multidisciplinary discussion involving thoracic surgeons, hematologists, radiation oncologists and interventional pulmonologists, with consideration of biopsy, radiotherapy, anti-CD20-based treatment and surveillance.

#### 3.5.6. Multidisciplinary Tumor Board & Clinical Decision-Making Pathway

Management of MTS requires a structured, multi-specialty workflow to balance urgent airway stabilization with long-term oncological control. The key roles across the care pathway are defined as follows:

- Radiology: Defines cross-sectional anatomical extent, tumor length, cartilage invasion, vascular relationships, and distant staging via CT, PET-CT, or MRI.
- Interventional Pulmonology/Thoracic Surgery: Performs diagnostic bronchoscopy and acute luminal restoration (debulking, laser, dilation, stenting) for symptomatic stabilization.
- Anesthesiology & Intensive Care (ICU): Secures advanced airway control in critical stenoses, manages perioperative ventilation/ECMO support, and oversees post-procedural monitoring.
- Medical & Radiation Oncology: Evaluates candidates for definitive or adjuvant chemoradiotherapy, systemic targeted agents, or palliative irradiation.

The phased decision-making pathway integrating these roles, from acute airway triage through definitive treatment and surveillance, is summarized in Figure 3.

#### 3.5.7. Conventional Surgical Approaches

Surgical approach is determined by anatomical location and lesion extent to ensure optimal exposure and control of adjacent structures [3,89].

- Median Cervical: Preferred for subglottic and upper cervical tracheal lesions via direct access [58].
- Partial Sternotomy/Right Thoracotomy: Required for lower tracheal and carinal lesions to manage the airway, carina, and mediastinum [12,19].
- Combined Cervico-Mediastinal: Used for extensive or lesions invading the mediastinum requiring complete tracheal exposure and vascular control during curative resections [12,90].

#### 3.5.8. Minimally Invasive Approaches

Video-assisted thoracoscopic surgery (VATS) and robotic surgery are emerging alternatives to open surgery in carefully selected patients with resectable tracheal or carinal tumors. Potential advantages include reduced chest wall trauma, postoperative pain, and hospital stay; however, these approaches remain technically demanding and require substantial surgical expertise [90,93,94].

Evidence supporting minimally invasive tracheal and carinal resections remains limited, with most available data derived from small retrospective series and highly selected patient populations. Technical feasibility depends on appropriate patient selection, tumor location and extent, surgical expertise, airway and ventilation management, and the ability to achieve a safe and reliable anastomosis [94,95].

- Thoracoscopic surgery: Thoracoscopic tracheal and carinal resections have been reported as technically feasible in selected patients, although their application remains limited by the complexity of airway reconstruction and the need for adequate oncological margins [93,94,95].
- Robotic surgery: Robotic approaches have also been described as feasible in selected cases; however, current evidence remains insufficient to establish oncological equivalence with conventional open surgery [95].

Despite increasing adoption of minimally invasive techniques, open surgery remains the established approach for complex tracheal and carinal resections, particularly when extensive exposure, vascular control, complex airway reconstruction, or maximal oncological clearance is required [3,28].

### 3.6. Postoperative Mortality and Outcomes

#### 3.6.1. Perioperative Mortality

Perioperative mortality following tracheal resection for MTS has decreased significantly in recent decades, driven by advancements in surgical technique, anesthesia, and perioperative care. In specialized high-volume centres, contemporary mortality rates are generally reported between 2% and 6% [4,96].

In the landmark series published by Grillo and Mathisen evaluating surgically managed primary tracheal tumors, perioperative mortality was below 5%, with the authors emphasizing the critical role of rigorous patient selection and center-specific surgical expertise [3,18]. Gaissert et al. reported results from the same institution [16,36,37]. In their single-institution retrospective series of 270 patients with tracheal ACC or SCC treated between 1962 and 2002, operative mortality among the 191 resected patients was 7.3%, a figure encompassing four decades of practice [16]. More recently, Ferreirinha et al. reported a perioperative mortality rate of approximately 2–3% in a contemporary single-center cohort that included both benign and malignant tracheal stenoses [97].

These findings demonstrate that while tracheal resection remains a complex procedure with inherent risk, perioperative mortality is acceptable when performed in high-volume institutions with dedicated airway surgery expertise [96,97].

#### 3.6.2. Postoperative Outcomes

Oncologic outcomes following tracheal resection for MTS are predominantly determined by resection radicality, histological subtype, and the appropriate administration of adjuvant therapy. Complete R0 resection with negative surgical margins is the primary determinant of long-term overall survival and local disease control [33,42,98].

Reported 5-year overall survival after resection varies widely across series (approximately 50–80% for ACC and 35–50% for SCC), reflecting differences in treatment era, patient selection, stage, margin status and adjuvant therapy; these figures should not be interpreted as precise, generalizable estimates (Table 4). In the largest single-institution series (Gaissert et al., *n* = 270; 191 resected), 5-year survival after resection was 52% for ACC and 39% for SCC, compared with 33% and 7.3%, respectively, in unresected patients [16]. Incomplete resections (R1 or R2 margins) are associated with local recurrence rates of 40–60%, underscoring the detrimental impact of positive margins on local control [6,16,44].

For ACC, several series indicate that microscopic positive (R1) margins may be partially mitigated by adjuvant radiotherapy without significantly compromising overall survival, owing to the indolent natural history and relative radiosensitivity of this histology. These observations, initially described by Grillo and Gaissert et al. [16,37], have been confirmed by modern studies evaluating adjuvant modalities in malignant tracheal tumors [16,32,43,50].

Ferreirinha et al. [97] corroborated these trends in a contemporary single-center cohort, demonstrating a 5-year overall survival rate of approximately 65–70% among patients treated with curative intent, with significantly superior outcomes observed in ACC compared to squamous cell carcinoma. The authors reaffirmed the essential roles of complete resection and stringent patient selection.

Postoperative radiotherapy is recommended in the setting of positive surgical margins or ACC with perineural invasion. Systemic chemotherapy plays a limited role in curative management and is primarily reserved for palliative intent in advanced squamous cell carcinoma. In carefully selected borderline resectable cases, neoadjuvant therapy may be considered to achieve tumor downstaging and increase the likelihood of complete R0 resection [28,32].

#### 3.6.3. Postoperative Complications

Complications occur in approximately 20% of patients after tracheal resection, about half of which are anastomotic [4]. In the largest reported series (Wright et al., 901 patients, predominantly with benign post-intubation stenosis and a minority with tumors), anastomotic complications occurred in 9%, including restenosis (4%), anastomotic separation (4%) and granulation tissue (1%), and were associated with a marked increase in mortality [4,59]. Factors associated with anastomotic failure included reoperation, diabetes, longer resections (≥4 cm), laryngotracheal resection and a pre-existing tracheostomy [59]. Among the 50 patients with neoplastic stenosis in the series by Piazza et al. (137 patients in total), complications occurred in 36%, and resection length and preoperative tracheotomy were associated with major surgical complications, while age over 70 years with cardiopulmonary comorbidities was associated with major medical complications [96]. Because most complication data derive from mixed or predominantly benign cohorts, rates in MTS may be higher, particularly after prior radiotherapy or extended resections. Major complications, their reported frequencies, risk factors, prevention and management are summarized in Table 5.

### 3.7. Functional Outcomes and Quality of Life

Following tracheal resection with primary anastomosis—including in resectable malignant cases—the literature consistently reports significant relief of respiratory symptoms and satisfactory recovery of ventilatory function. In most series, patients achieve an acceptable quality of life, contingent upon rigorous patient selection, the execution of a tension-free anastomosis, and diligent postoperative bronchoscopic surveillance [4,59].

In the study by Timman et al. [101], which included patients with acquired benign laryngotracheal stenosis and therefore provides only indirect evidence for MTS, postoperative functional evaluation demonstrated significant improvement in quality of life compared with preoperative baseline, accompanied by a high overall success rate. However, the authors noted that laryngotracheal resections are associated with a higher incidence of airway complications than segmentary tracheal resections. In the analysis published by Piazza et al., which included 50 patients with neoplastic pathology among 137 cases, the decannulation rate reached approximately 96% in the malignant group, corroborating that restoring a stable, cannula-free airway is achievable in high-volume centres [96].

The review by Auchincloss and Wright in the Journal of Thoracic Disease emphasizes that the majority of patients undergoing tracheal or laryngotracheal reconstruction recover effective respiration and satisfactory daily functional capacity [4,59].

In malignant pathology, functional benefits—particularly the alleviation of dyspnea—can be substantial when resection is performed with curative intent and airway continuity is restored. Nevertheless, long-term outcomes remain dependent on histological subtype, surgical margin status, and the necessity of adjuvant therapy [6,16,31,44].

In conclusion, tracheal resection with primary anastomosis remains the gold-standard curative treatment for resectable malignant tracheal stenosis, offering superior oncologic and functional outcomes when performed in specialized centres [17,33,42]. Achieving complete R0 resection with negative margins is the primary prognostic factor for long-term survival and local disease control [6,7,16,44]. Furthermore, the integration of modern intraoperative ventilation strategies and extracorporeal life support may extend operability in selected critical stenoses and complex carinal lesions treated in experienced centers [11,80]. Adjuvant radiotherapy plays a crucial role in consolidating local control, particularly in patients with ACC presenting with microscopic positive (R1) margins [32,50]. Overall, surgical management must be individualized, multidisciplinary, and centralized, adhering strictly to established technical principles of airway surgery [4,6,18,44,58].

### 3.8. Interventional and Palliative Management

Interventional and palliative management is indicated when complete surgical resection is unachievable due to advanced loco-regional extension, distant metastases, or severe patient comorbidities. The primary objective is to restore and maintain airway patency, thereby alleviating dyspnea, preventing asphyxiation, and improving quality of life [22,29,65].

The therapeutic strategy is phased and tailored to the severity of the obstruction, the underlying mechanism of the stenosis, and overall oncologic status. The immediate priority is rapid airway recanalization via interventional BC, frequently followed by tracheal stenting to preserve luminal patency. Following respiratory stabilization, patients may proceed to radiotherapy, chemotherapy, or other systemic oncologic treatments [2,22,46].

#### 3.8.1. Interventional Bronchoscopy

Interventional BC is the modality of choice for airway recanalization in advanced malignant tracheal stenosis, serving a palliative, therapeutic, or preoperative stabilizing role. It is indicated for significant intraluminal obstruction, unresectable stenosis, and restenosis following prior interventions [24,65,77].

- Mechanical Debulking: Mechanical debulking allows for the direct excision of tumor mass and immediate recanalization of the tracheal lumen. Its primary advantage is the rapid relief of respiratory symptoms; however, the procedure is limited by bleeding risks and tumor recurrence. In a single-center series of central airway tumors (a mixed CAO population, not restricted to MTS), technical success rates between 88% and 100% were reported [102].
- Laser Therapy and Electrocautery: Laser modalities, including Neodymium-doped Yttrium Aluminum Garnet and diode lasers, enable vaporizing tumor tissue with effective hemostasis and immediate relief of obstruction. Electrocautery and argon plasma coagulation (APC) are valuable for exophytic lesions and are frequently combined with stenting. Series of mixed malignant central airway obstruction (not restricted to MTS) report recanalization rates of approximately 80%, with significant clinical improvement in most patients [20,103,104].
- Cryotherapy: Cryotherapy induces tumor necrosis through repeated freeze–thaw cycles. Although its action is slower compared with laser ablation, it is particularly useful in friable lesions and high-bleeding-risk scenarios [105]. Inaty et al. [106] reported restoration of airway patency in approximately 95% of patients in a single-center series of airway obstruction of various etiologies.
- Photodynamic Therapy (PDT): PDT employs photosensitizers locally activated by light and has selective indications, predominantly in superficial tumors or lesions with submucosal extension [107]. Yang et al. [108] reported an objective response rate of approximately 77% in a two-center retrospective series of tracheal ACC.

Overall, endoscopic techniques offer rapid symptom relief, though their effects may be transient, often requiring repeated interventions or combination with stenting and systemic oncologic therapy [22,107].

#### 3.8.2. Selection of Bronchoscopic Modalities and Integrated Treatment Algorithm

The choice among bronchoscopic modalities depends on obstruction morphology (endoluminal, intramural, extrinsic or mixed), lesion vascularity, the degree and length of obstruction, the presence of a fistula, expected survival and treatment intent [2,22,30,73]. Most of the underlying evidence derives from mixed malignant CAO cohorts and expert recommendations rather than from MTS-specific studies. A practical, phased approach distinguishes four steps:

- Emergency airway stabilization: securing ventilation with rigid BC, with ECMO standby or support in selected critical cases, and immediate core-out of the dominant endoluminal component.
- Tumor debulking: mechanical debulking combined with thermal ablation (laser, electrocautery/APC) for endoluminal lesions; cryorecanalization or APC for friable or bleeding-prone tumors. Delayed-effect modalities (PDT, conventional cryotherapy) are unsuitable in critical obstruction.
- Dilation and stenting: balloon or rigid dilation followed by stenting for extrinsic compression and mixed lesions, and after debulking when significant residual obstruction persists or rapid regrowth is expected. Stents should generally be avoided in potentially resectable disease, as they may compromise subsequent resection [3].
- Definitive oncological treatment: resection when feasible after re-staging, or radiotherapy and/or systemic therapy for unresectable disease, followed by bronchoscopic surveillance.

Figure 3 outlines the multidisciplinary decision-making algorithm from initial triage to surveillance, while Table 6 summarizes modality selection based on airway morphology and clinical presentation. 

#### 3.8.3. Tracheal Stents

Tracheal stenting aims to maintain airway patency following recanalization or in stenoses caused by extrinsic compression and structural instability of the tracheal wall [20,22,109].

The earliest widely used devices were the T-tubes described by Montgomery [110], followed by Dumon-type silicone stents, which standardized the concept of endoluminal airway stenting [23,111]. Subsequently, the development of self-expanding metallic stents (SEMS) made of nitinol expanded therapeutic indications, particularly for extrinsic compression and complex stenoses [24,112]. A detailed comparison of indications, contraindications, and complication profiles across silicone, metallic, hybrid, Y-stents, and bioresorbable devices is summarized in Table 7.

Primary indications include unresectable MTS resulting from advanced local tumor extension, systemic disease, or severe comorbidities that contraindicate major surgical intervention [22,24].

Stenting is also recommended for critical stenoses that persist or recur following endoscopic debulking procedures when achieved recanalization cannot be maintained through conservative means [24,46].

A major indication is represented by malignant tracheoesophageal fistulas, in which covered tracheal stents—sometimes combined with esophageal stents—allow for the effective separation of the respiratory and digestive tracts [115]. Because the goal in fistula management is sealing and separation of the aerodigestive tracts rather than luminal restoration alone, stent selection follows different principles from routine malignant stenosis and is discussed separately in Section 3.9.

Tumor recurrence following surgical resection or radiotherapy that leads to symptomatic tracheal obstruction represents another common scenario in which stenting offers a significant palliative benefit. Additionally, complex carinal or tracheobronchial obstructions can benefit from specially configured devices, including Y-stents [22,70,116].

#### 3.8.4. Bioresorbable and Bioprosthetic Stents

Bioresorbable stents represent a recent technological advancement aimed at mitigating the long-term complications associated with permanent endoluminal devices. Initial clinical applications primarily utilized polydioxanone and poly-L-lactic acid [113,117].

Lischke et al. [118] published one of the early clinical series evaluating polydioxanone stents in benign bronchial stenoses after lung transplantation (not MTS), reporting preserved luminal patency for approximately 8–12 weeks and an initial clinical success rate of 75–80%, notably without requiring device removal.

Subsequent experimental studies and small clinical series published between 2013 and 2023, including reports by Freitag [70], Zopf [119], and Aravena [25]—demonstrated the technical feasibility of patient-customized bioresorbable stents fabricated via three-dimensional (3D) printing. These devices maintained tracheal lumen stability for 2–3 months, and lower rates of migration and infectious complications than permanent metallic or silicone stents have been suggested. However, these observations derive from experimental models, individual cases and small, heterogeneous, predominantly benign series without direct comparative studies, and should be regarded as hypothesis-generating rather than as evidence of superiority [113,114].

However, in malignant tracheal stenosis, the application of bioresorbable devices remains exceptional, with only isolated reports available. Current indications are restricted to highly selected cases, particularly in patients with limited life expectancy or as a temporary bridge pending a therapeutic response to radiotherapy or systemic chemotherapy [113].

In conclusion, although preliminary data suggests a favorable complication profile and promising clinical efficacy, bioresorbable and bioprosthetic stents should currently be considered investigational in MTS. Large-scale prospective and comparative studies are needed to clearly define their specific indications, safety profile, and definitive role in the management of malignant tracheal stenoses [113,114].

#### 3.8.5. Extracorporeal Support

ECMO represents an advanced life-support modality utilized in critical tracheal stenoses when conventional mechanical ventilation cannot be safely maintained. This approach is indicated in critical airway stenoses where extreme luminal narrowing poses a risk of complete airway collapse during induction, interventional BC, or surgical resection. Initiation of extracorporeal support maintains oxygenation and carbon dioxide elimination independently of airway patency, thereby mitigating the risks of severe hypoxia and cardiorespiratory arrest [11,79].

ECMO is likewise valuable in complex “airway-off” interventions, such as extensive tracheal resections or carinal reconstructions, where airway continuity is temporarily interrupted. In these settings, the technique provides a stable, unobstructed operative field, facilitating safe tracheobronchial anastomoses. Another major indication includes the inability to maintain mechanical ventilation via natural airways, attributable either to complete/near-complete obstruction or to tumor-induced or post-therapeutic anatomical distortion [11,120].

The series by Hong et al. [120], which included patients with central airway obstruction of various etiologies, suggested that ECMO utilization in specialized centres reduces the risk of intraoperative hypoxia and enables the safe execution of complex airway procedures.

The implementation of ECMO in malignant tracheal stenoses should be restricted to high-volume multidisciplinary centres following a careful risk-benefit assessment integrated into an individualized therapeutic plan [79,81]. ECMO should therefore be regarded as an established rescue or adjunctive support strategy for selected critical airway procedures in experienced centers, rather than as a component of routine management; the supporting evidence consists of case series and institutional experience rather than comparative studies.

#### 3.8.6. Palliative Radiotherapy and Chemotherapy

External beam radiation therapy (EBRT) and systemic modalities—including chemotherapy and targeted oncologic agents—are frequently integrated into the palliative strategy for malignant tracheal stenoses. These may serve as primary modalities when endoscopic interventions are contraindicated, or as complementary therapies following endoscopic recanalization and stenting to control local disease progression, alleviate symptoms, and preserve airway patency [65].

Historical data regarding palliative irradiation of tracheal neoplasms indicate significant symptomatic benefit. In the series published by Makarewicz et al. [121], radiotherapy administered as monotherapy achieved favorable clinical palliation in approximately 75% of patients. In slower-growing glandular subtypes, particularly unresectable tracheobronchial ACC, an analysis by Levy et al. [122]. reported objective tumor response rates of approximately 87% following radiotherapy.

Regarding the combination of endoscopic recanalization, stenting, and oncologic therapies, Mallow et al. [123] reported, in a retrospective cohort of malignant airway obstruction not restricted to the trachea, that EBRT combined with airway stenting was associated with superior survival compared to stenting alone. In real-world clinical practice, a series by Leelayuwatanakul et al. [124] reported a median post-stent survival of 97 days, with nearly half of the cohort receiving chemotherapy and/or radiotherapy, reflecting the routine application of systemic or loco-regional treatment following airway stabilization.

Overall, radiotherapy plays an essential palliative role in symptom control and local progression delay, while systemic therapy is primarily utilized in advanced SCC and disseminated disease. Integrating these therapies post-debulking or stenting can prolong the time to restenosis and improve clinical outcomes in appropriately selected patients [122,123,124].

### 3.9. Malignant Tracheoesophageal Fistulas

Malignant tracheoesophageal fistulas represent a severe complication of upper aerodigestive tract malignancies, arising from simultaneous erosion of the esophageal and tracheal walls caused by direct tumor invasion or, less frequently, radiation-induced necrosis of fragile, hypovascularized tissue. These are predominantly encountered in esophageal carcinoma and central or apical lung cancers, with an estimated incidence of 5% to 15% in advanced stages [125,126].

The clinical presentation is characteristic, featuring paroxysmal coughing upon deglutition, recurrent aspiration pneumonia, fetid breath, and rapidly progressive weight loss. Diagnosis is suggested by contrast-enhanced CT, which can visualize the aerodigestive communication and associated pulmonary complications, and is confirmed via BC and esophagoscopy to establish the exact location and dimensions of the fistula [126,127].

Management is predominantly palliative, with the primary objective of separating the respiratory and digestive tracts to prevent aspiration [126]. Subsequently, Wassermann et al. [128,129] reported superior outcomes using dual tracheoesophageal stenting, resulting in reduced recurrent pneumonia and improved quality of life.

Similar findings were confirmed by Shin et al. in a large cohort of patients with malignant aerodigestive fistulas, highlighting the short-term efficacy of covered stents in symptom palliation. Freitag et al. [70,116] underscored the importance of selecting the appropriate stent design based on the fistulous mechanism and the patient’s clinical status.

Temporary enteral nutrition and palliative radiotherapy may be combined to reduce tumor burden and alleviate symptoms. However, overall prognosis remains guarded, as malignant tracheal fistulas serve as a clinical marker of end-stage disease [115,125,126,127].

Stent strategy in malignant fistula differs from that in routine MTS: covered stents are required, and the choice between airway, esophageal or double stenting depends on fistula level, coexisting airway narrowing and the risk that an esophageal stent compresses the trachea. Airway stenting is generally performed first, or concurrently, when airway compromise is present or anticipated, and Y-shaped covered stents may be needed for fistulas close to the carina [115,125,126].

## 4. Discussion

### 4.1. Current Challenges in MTS Management

MTS is a rare entity, which significantly limits the feasibility of randomized controlled trials. Available evidence derives predominantly from retrospective series, observational studies, and multicenter reports, lowering the overall level of evidence and complicating inter-center outcome comparisons [5,6,7].

Disease heterogeneity represents another major obstacle, and this variability hinders the development of standardized recommendations and uniform clinical practice guidelines [5].

A further limitation is that much of the evidence on bronchoscopic interventions, stenting, extracorporeal support and postoperative complications derives from mixed CAO cohorts or from benign stenoses, whereas specific thresholds for resection length, technical success, survival and recurrence originate largely from small retrospective series from highly specialized centers. These data should be interpreted in the context of patient selection, histology, stage, resection status and adjuvant treatment, and should not be extrapolated from epithelial tumors to tracheal lymphomas or other rare neoplasms.

At present, there is no consensus regarding the precise anatomical and functional criteria defining tracheal resectability. The ideal length considered safe for resection ranges between 4 and 6 cm, depending on institutional expertise, patient age, tissue compliance, and surgical release techniques employed. Although the advent of ECMO and laryngotracheal release maneuvers has expanded these boundaries, robust data regarding long-term safety and outcomes remain limited [130].

Patient outcomes are highly dependent on institutional volume and expertise. Units with established specialization in airway surgery report perioperative mortality rates below 3%, whereas higher mortality has been reported in less experienced settings. Although centralizing care in dedicated reference centres is consistently advocated in the literature, global implementation remains uneven due to logistical, geographical, and socioeconomic barriers [96].

### 4.2. Emerging Technological Innovations

In this review, technologies are categorized according to their level of clinical readiness: established (routine use supported by clinical series and guidelines, e.g., R0 resection, bronchoscopic recanalization and stenting); established in selected settings (e.g., ECMO in experienced centers); early clinical (feasibility shown in small human series, e.g., stented aortic matrices and patient-specific 3D-printed stents); investigational (e.g., bioresorbable airway stents in MTS and AI-based tools); and experimental or preclinical (e.g., 3D bioprinting and drug-eluting biodegradable stents).

#### 4.2.1. Bioengineering and Regenerative Surgery

Advances in three-dimensional (3D) bioprinting and regenerative surgery offer novel avenues for personalized airway reconstruction, aiming to produce tracheal substitutes that provide mechanical stability, luminal patency, biological integration, and tissue remodeling. Three-dimensional bioprinting, however, remains experimental and limited to preclinical models, whereas stented aortic matrices have reached early clinical evaluation [10,64,131,132,133].

Experimental models utilizing polymeric scaffolds and cellular constructs have demonstrated technical feasibility:

- Bae et al. & Park et al. [131,133]: reported, in preclinical in vitro and animal studies, 3D-bioprinted artificial tracheal models using epithelial and mesenchymal stem cells, showing promising mucosal epithelialization and graft integration.
- Martinod et al. (JAMA & TRITON-01 trial) [134]: in a prospective single-center study of 35 adult patients (TRITON-01), demonstrated the feasibility of airway replacement using stented aortic matrices, reporting encouraging 2- and 5-year survival rates, despite stent-related complications and re-intervention requirements.
- Drug-Eluting & Custom Stents: Chao et al. [117] showed, in in vitro and animal studies, that biodegradable, cisplatin-eluting stents combine mechanical support with sustained local antineoplastic activity. Patient-specific 3D-printed stents match individual airway geometry, mitigating device-airway mismatch, though clinical evidence remains limited to small series.

#### 4.2.2. Artificial Intelligence and Augmented Bronchoscopy

Integrating artificial intelligence (AI) into thoracic imaging and BC may, in the future, enhance diagnostic precision and procedural planning in MTS; current applications remain investigational:

- Charbonnier et al. [135]: developed convolutional-network-based automated airway segmentation on CT, a methodological study not specific to MTS that may support tracheal morphometric analysis.
- Chen et al. [136]: reviewed emerging deep-learning algorithms for automated recognition of airway anatomical structures during endoscopy and intubation, most of which have been evaluated only in retrospective or experimental settings.

At present, these AI applications have been evaluated only in retrospective or experimental datasets, largely derived from mixed airway pathology rather than from MTS, without prospective external validation or demonstrated impact on clinical outcomes; they should therefore be regarded as investigational tools rather than established components of clinical decision-making.

### 4.3. Future Directions in MTS Management

#### 4.3.1. Bioengineered Tracheal Reconstruction

Stented aortic allografts and autologous stem cell-seeded 3D bioprinting represent early clinical and experimental approaches, respectively, that may in the future enable regenerative airway replacement [137]. Widespread clinical translation requires standardization of biofabrication processes, mitigation of infectious risks and proof of long-term functional stability through prospective multicenter trials [138].

In pilot centers, patient-specific 3D-printed models and custom stents are being used on an individual basis for preoperative planning and complex stenting, although the evidence is limited to case reports and small series [25,26].

#### 4.3.2. Integrated Multimodal Therapy

Modern MTS management centers on structured, multidisciplinary oncology workflows involving thoracic surgeons, interventional pulmonologists, radiation oncologists, medical oncologists, and intensivists [91,92].

Sequential therapeutic protocols—initiating with rapid endoscopic recanalization and stenting to stabilize ventilation, followed by targeted chemoradiotherapy—have been associated with improved local disease control and survival in retrospective series in advanced or initially unresectable disease [139].

Furthermore, long-term evidence from tertiary thoracic surgery centers highlights that survival and disease recurrence in central respiratory malignancies are fundamentally shaped by tumor T-stage, margin status, specific histomorphological characteristics (such as cell arrangement and tumor budding in squamous cell carcinoma), and coexisting non-oncological pulmonary complexities [140,141,142].

## 5. Conclusions

A review of six decades of literature yields key insights regarding malignant tracheal stenosis. Despite its low incidence, this condition presents a severe clinical burden on vital respiratory function. Adenoid cystic carcinoma and squamous cell carcinoma represent the primary histological subtypes, differing fundamentally in biological behavior, aggressiveness, and therapeutic response. Management demands a multimodal diagnostic strategy that integrates clinical evaluation, advanced cross-sectional imaging, and endoscopic tissue confirmation to guide treatment planning.

Complete surgical resection with negative margins remains the gold standard curative therapy for resectable cases. Minimally invasive approaches (VATS or robotic surgery) are not established alternatives to open surgery; they may be considered only in highly selected patients treated in experienced airway centers, as supporting data are limited to small retrospective series. In advanced or unresectable diseases, interventional and palliative modalities form the cornerstone of management. Tracheal stenting, endoscopic ablation with laser or electrocautery, and mechanical debulking enable rapid restoration of airway patency and improve quality of life.

R0 resection for appropriately selected resectable disease and interventional airway management for unresectable symptomatic disease are supported substantially more strongly than any other approach, although the evidence still consists predominantly of retrospective series from specialized centers. ECMO is an established rescue or adjunctive support strategy in selected critical airway procedures performed in experienced centers, but not a component of routine management. By contrast, three-dimensional bioprinting and tissue-engineered reconstruction (experimental or early clinical), bioresorbable stents and AI-based diagnostic or decision-support tools (investigational) require prospective validation before clinical adoption. Rare entities such as tracheal lymphomas and tumor-like disorders require distinct diagnostic and therapeutic pathways, and international collaborative registries are needed to generate stronger evidence and standardized guidelines for this rare pathology.

## Figures and Tables

**Figure 1:**
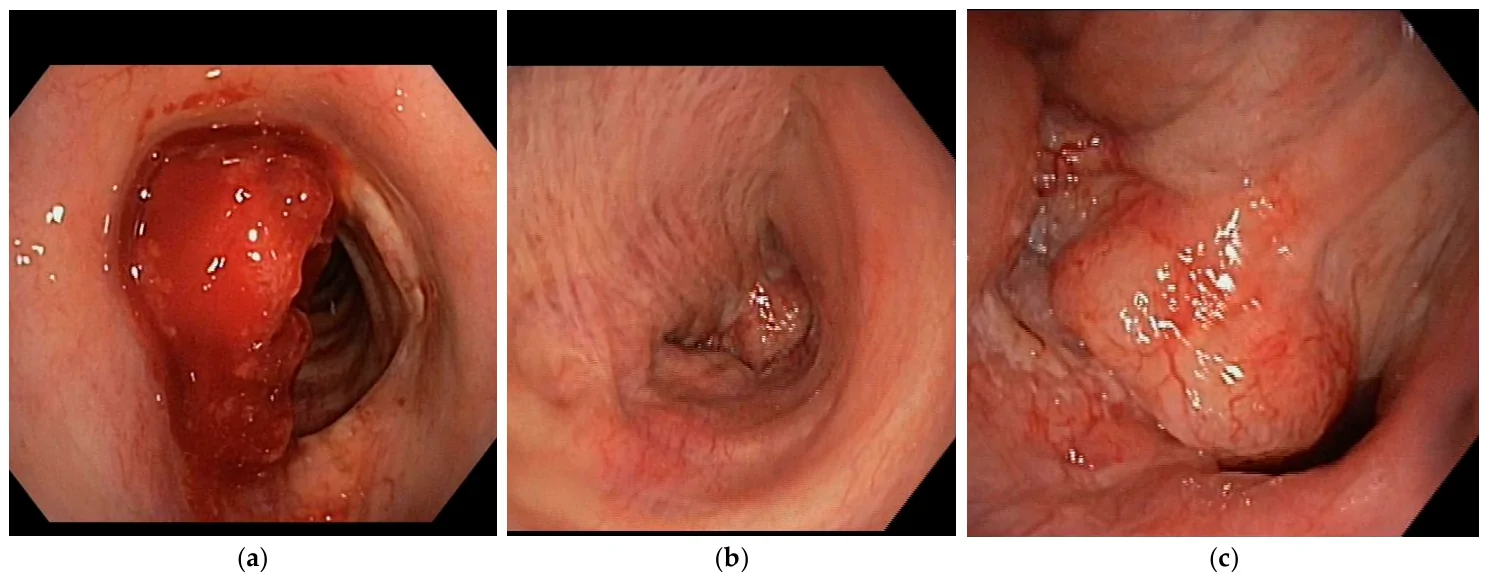
Endoscopic view of MTS. (**a**) Endobronchial view showing a hypervascular, friable exophytic endotracheal tumor causing subtotal luminal occlusion; (**b**) Severe tracheal obstruction secondary to circumferential mucosal infiltration and luminal narrowing; (**c**) Endoscopic view of an exophytic tumor mass causing subtotal tracheal stenosis.

**Figure 2:**
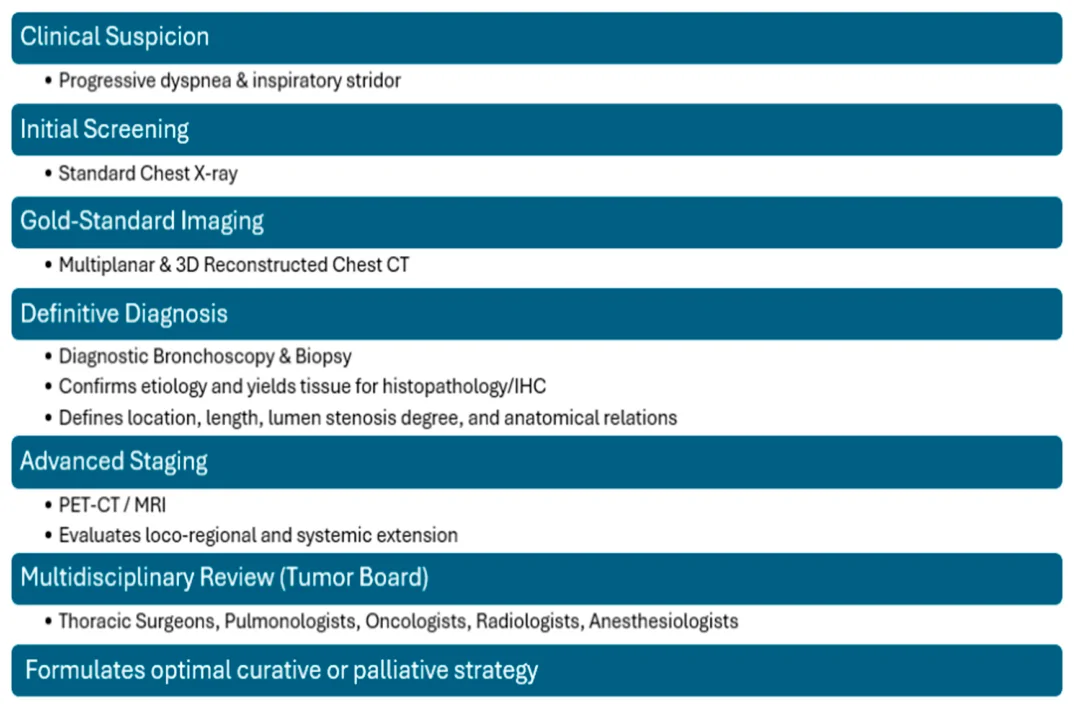
Diagnostic algorithm.

**Figure 3:**
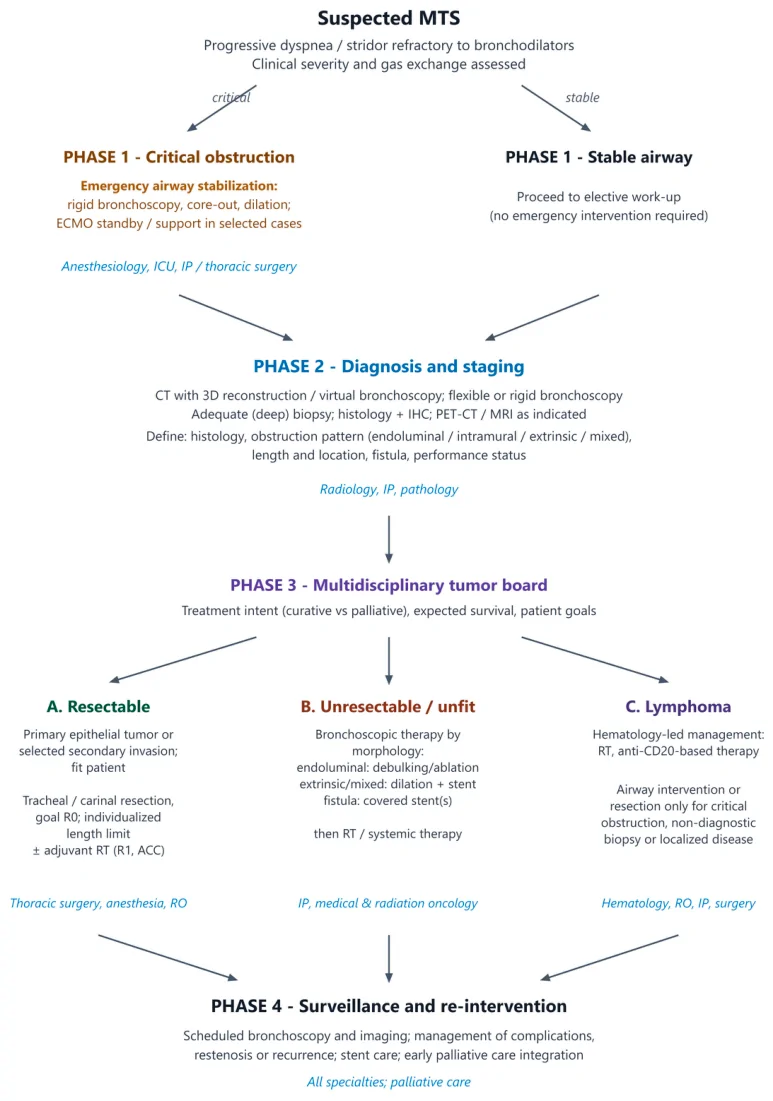
Proposed multidisciplinary decision-making pathway for malignant tracheal stenosis, from acute airway triage to definitive treatment and surveillance, with the specialties involved at each phase. The pathway is a synthesis proposed by the authors based on the cited literature and expert recommendations [2,22,28,30,91,92], not a validated algorithm. IHC, immunohistochemistry; IP, interventional pulmonology; RO, radiation oncology; RT, radiotherapy.

**Table 1:** Anatomic and functional classification used for surgical planning and resectability assessment. This is a pragmatic, author-derived surgical planning scheme based on established principles of tracheal surgery [3,4,58,59], not a formally validated classification; the length ranges are indicative, as resectability also depends on patient age, tissue compliance, prior radiotherapy and the release maneuvers available.

Type	Primary Location	Anatomical Features	Preferred Surgical Approach
I	Subglottic/Cricotracheal	<2 cm distal to vocal cords	Cricotracheal resection + thyrotracheal anastomosis
II	Cervical Trachea	2–4 cm, no carinal invasion	Simple segmental resection
III	Upper thoracic trachea	4–6 cm, limited carina involvement	Thoracic resection and release manoeuvres
IV	Carinal/Tracheobronchial	Involves carina/main bronchi	Complex carinal resection/Y-stenting

**Table 2:** Clinico-pathological categories of malignant tracheal stenosis and their treatment implications [2,6,16,44,49,56,60,62]. ACC, adenoid cystic carcinoma; MALT, mucosa-associated lymphoid tissue; RT, radiotherapy; SCC, squamous cell carcinoma.

Category (Examples)	Typical Obstruction Pattern	Biology/Prognosis	Role of Surgery	Role of Bronchoscopy/Stenting	Role of Oncological Therapy
Primary epithelial: ACC	Intramural with submucosal and perineural longitudinal spread; endoluminal component	Indolent; late recurrences; relatively radiosensitive	Resection of choice when feasible; R1 acceptable if R0 not achievable	Bridge to surgery or palliation; avoid stents if resectable	Adjuvant RT for positive margins/perineural invasion; definitive RT if unresectable
Primary epithelial: SCC	Predominantly endoluminal; may be mixed	Aggressive; tobacco-related; nodal spread	Resection for localized disease	Debulking/ablation ± stent for palliation	Adjuvant or definitive (chemo)radiotherapy; systemic therapy in advanced disease
Other primary epithelial (mucoepidermoid, neuroendocrine)	Mostly endoluminal, polypoid	Low-grade forms favorable; high-grade forms aggressive	Resection	Endoscopic resection in selected low-grade polypoid lesions	Limited; histology-specific
Primary lymphoma (e.g., MALT)	Diffuse submucosal/circumferential thickening; mimics benign stenosis	Indolent; radio- and chemosensitive	Not a standard; selected cases only (critical obstruction, non-diagnostic biopsy, localized disease)	Airway stabilization; deep biopsy	RT, anti-CD20-based therapy; hematology-led
Secondary: direct invasion (thyroid, esophagus, lung)	Intramural, extrinsic or mixed; possible fistula	Depends on primary; poor for esophageal and lung cancer	Selected en bloc resection, mainly differentiated thyroid carcinoma	Frequently required: debulking + stent; covered stents for fistula	Primary tumor-directed (chemo)radiotherapy and systemic therapy
Secondary: metastatic (hematogenous, nodal) and mediastinal lymphoma	Endoluminal (hematogenous) or extrinsic (nodal, lymphoma)	Generally poor (except responsive lymphomas)	Rarely indicated	Palliative recanalization/stenting for symptomatic obstruction	Systemic therapy ± RT

**Table 3:** Clinical manifestations in MTS.

Symptom	Characteristics
Progressive dyspnea	Initially exertion dyspnea, then rest dyspnea
Inspiratory stridor	Suggests severe stenosis
Irritative Cough	Frequently persistent
Haemoptysis	Suggests tumor invasion
Dysphagia	Suggest esophageal involvement
Dysphonia	Suggests recurrent laryngeal nerve involvement

**Table 4:** Reported survival and recurrence after treatment of primary tracheal malignancies according to histology, with study population and treatment context. Figures derive from retrospective series spanning different treatment eras and selection criteria and should not be interpreted as precise or directly comparable estimates. ACC, adenoid cystic carcinoma; MALT, mucosa-associated lymphoid tissue; RT, radiotherapy; SCC, squamous cell carcinoma.

Histology	Source and Study Design	Population and Treatment Context	5-Year Overall Survival	Local Recurrence/Comments
ACC	Gaissert et al. [16]; single-center retrospective (USA), 1962–2002	135 ACC (of 270 ACC/SCC); resection with or without postoperative RT; airway margins positive in 59% of resected ACC	52% resected; 33% unresected (10-year: 29% resected)	Late recurrences characteristic; complete resection and negative margins prognostic
ACC	Maziak et al. [32]; single-center retrospective (Canada), 1963–1995	38 ACC; 32 resected (26 with adjuvant RT), 6 RT alone	Mean survival 9.8 years (complete resection), 7.5 years (incomplete), 6.2 years (RT alone)	Hematogenous metastases in 44%; microscopic extension beyond gross tumor limits
SCC	Gaissert et al. [16]; single-center retrospective (USA), 1962–2002	135 SCC (of 270); resection with or without postoperative RT; airway margins positive in 18% of resected SCC	39% resected; 7.3% unresected (10-year: 18% resected)	Nodal metastases frequent; outcome strongly dependent on complete resection
Mixed primary tracheal malignancies	Webb et al. [42]; single-center retrospective (USA), 1945–2004	74 patients (SCC 46%, ACC 26%, other 28%); surgery ± postoperative RT vs. RT alone	5-year all-cause mortality 79.3% for the whole cohort; better outcomes for ACC and cervical tumors	Heterogeneous histology and treatment eras
Mucoepidermoid carcinoma	Hsieh et al. [99]; single-center retrospective	41 surgically treated pulmonary (predominantly bronchial) mucoepidermoid carcinomas; indirect evidence, not tracheal-specific	57.9%	Recurrence 39%; grade-dependent
Primary tracheal lymphoma (e.g., MALT)	Kusunoki et al. [100]; case reports and literature reviews [49,62]	Isolated cases; resection, bronchoscopic therapy, RT, rituximab-based therapy	Not estimable	Indolent; outcomes not comparable with epithelial tumors
Sarcomas and other rare tumors	Small institutional series and reviews [6,36]	Very small, heterogeneous cohorts	Not reliably estimable	Histology-specific behavior

**Table 5:** Main complications after tracheal resection and reconstruction: reported frequencies, risk factors, prevention and management. Frequencies derive predominantly from mixed cohorts in which benign stenoses predominate (e.g., Wright et al., *n* = 901) and may underestimate the risk in MTS [4,59,96].

Complication	Reported Frequency	Main Risk Factors	Prevention	Management
Anastomotic separation/dehiscence	~4% (all anastomotic complications 9%) [59]	Excessive tension, long resection (≥4 cm), diabetes, reoperation, prior radiotherapy, preoperative tracheostomy	Tension-free anastomosis; release maneuvers; limited circumferential dissection; neck flexion (guardian suture); tissue flap coverage in high-risk fields	Secure airway; bronchoscopic assessment; minor: conservative care; major: T-tube or tracheostomy, flap coverage or re-operation [4]
Anastomotic restenosis	~4% [59]	Tension, ischemia, granulation tissue, reoperation	As above; absorbable sutures; minimal devascularization	Bronchoscopic dilation or laser; stent or T-tube; re-resection for durable relief [4]
Granulation tissue	~1% [59]	Non-absorbable sutures; infection	Absorbable sutures	Bronchoscopic removal; local steroids [4]
Recurrent laryngeal nerve injury/hoarseness	Nerve injury ~1.5%; hoarseness ~5% [4]	Laryngotracheal or cervical resections, paratracheal dissection, reoperation	Dissection close to the tracheal wall; nerve identification	Observation and voice therapy; tracheostomy if bilateral palsy compromises the airway
Laryngeal edema/dysphagia and aspiration	Impaired swallowing 2–4% [4]	Subglottic and laryngotracheal resections; suprahyoid release	Careful release; perioperative steroids when indicated	Steroids, diuretics, nebulized epinephrine; swallowing rehabilitation; re-intubation or tracheostomy if severe [4]
Pulmonary complications (pneumonia, atelectasis, respiratory failure)	Variable; main source of medical morbidity [96]	Age > 70 years, cardiopulmonary comorbidity, aspiration	Early extubation; physiotherapy; bronchoscopic toilet	Antibiotics, bronchoscopic toilet, ventilatory support
Wound infection	3–10% [4]	Diabetes, pre-existing tracheostomy, reoperation	Antibiotic prophylaxis; meticulous closure	Drainage and antibiotics
Tracheo-innominate or tracheoesophageal fistula	Rare (3/901 each) [4,59]	Anastomotic separation, infection, extensive dissection	Tissue interposition between anastomosis and artery/esophagus	Tracheo-innominate: emergency control and arterial ligation; tracheoesophageal: diversion and delayed repair [4]

**Table 6:** Selection of bronchoscopic modalities in malignant tracheal stenosis according to obstruction morphology and clinical scenario, based largely on mixed malignant central airway obstruction data and expert recommendations [2,22,24,30,73,107]. APC, argon plasma coagulation; BC, bronchoscopy; MDT, multidisciplinary team; PDT, photodynamic therapy; RT, radiotherapy; SEMS, self-expanding metallic stent.

Morphology/Clinical Scenario	Preferred Modalities	Onset of Effect	Comments and Cautions
Endoluminal (exophytic), critical obstruction	Rigid BC core-out and mechanical debulking; laser or electrocautery/APC for hemostasis	Immediate	First-line in emergency; ensure ventilation control
Endoluminal, hypervascular or friable	APC/electrocautery, laser photocoagulation, cryorecanalization	Immediate	High bleeding risk; rigid BC preferred
Intramural/infiltrative (e.g., ACC)	Limited debulking + dilation ± stent; radiotherapy	Immediate (dilation)/delayed (RT)	Ablation alone ineffective; submucosal extent often underestimated
Extrinsic compression	Dilation + stent (silicone or covered SEMS)	Immediate	Ablation not indicated (no endoluminal target)
Mixed	Debulking of the endoluminal component + stent	Immediate	Most frequent pattern in secondary MTS
Tracheoesophageal fistula	Covered airway stent ± esophageal stent (sequence decided by the MDT)	Immediate	Aim is sealing, not only luminal restoration (Section 3.9)
Superficial or non-critical lesion, poor surgical candidate	PDT, cryotherapy	Delayed (days)	Not for critical obstruction; requires follow-up bronchoscopic toilet
Potentially resectable disease	Diagnostic BC; limited core-out only as a bridge to surgery	–	Avoid stents where possible (may compromise resection)
Limited life expectancy	Least invasive option providing immediate relief	Immediate	Align with patient goals; early palliative care

**Table 7:** Comparison of tracheal stent types used in malignant tracheal stenosis: indications, limitations and complication profiles. Relative frequencies are qualitative and derive mainly from mixed malignant central airway obstruction series [21,22,23,24,46,109,113,114]. BC, bronchoscopy; MTS, malignant tracheal stenosis; SEMS, self-expanding metallic stent.

Feature	Silicone (Tubular/Studded)	Covered SEMS (Nitinol)	Hybrid	Y-Stent (Silicone or Metallic)	Bioresorbable
Typical indications	Endoluminal or mixed obstruction after debulking; extrinsic compression; bridge to oncological response	Extrinsic compression, irregular or tortuous airway; patients unfit for rigid BC; fistula	Extrinsic or mixed obstruction requiring conformability with removability	Carinal/tracheobronchial involvement; fistula close to the carina	Investigational in MTS; temporary support in highly selected cases
Contraindications/limitations	Requires rigid BC and general anesthesia; poor fit in irregular or conical airways	Uncovered SEMS unsuitable when removal may be needed; avoid in potentially resectable disease	Limited availability; high cost	Technically demanding insertion; secretion management	Short duration of support; very limited MTS data
Migration	Relatively frequent (especially in extrinsic compression)	Less frequent	Low to moderate	Low (anchored at the carina)	Not well defined
Granulation tissue	Mainly at stent ends; relatively low	Frequent at stent ends; tumor ingrowth through uncovered segments	Mainly at stent ends	At the limbs and stent ends	Reported; limited data
Secretion retention/infection	Common (impaired mucociliary clearance)	Less than silicone; infection possible	Intermediate	Frequent, particularly within the limbs	Limited data
Fracture	Not applicable (deformation possible)	Metal fatigue fracture with long dwell times	Possible	Rare with silicone	Fragmentation during degradation
Removal	Easy with rigid BC	Difficult after epithelialization; should be considered permanent	Generally feasible	Feasible with rigid BC (silicone)	Not required (degradation); repeated placement may be needed
Intrinsic vs. extrinsic obstruction	Both; preferred when later removal is anticipated	Especially extrinsic and mixed	Extrinsic and mixed	Both, at the carina	Mainly benign stenoses in published series

## Data Availability

No new data were created or analyzed in this study. Data availability is not applicable to this article.

## Abbreviations

3D	Three-Dimensional
ACC	Adenoid Cystic Carcinoma
AI	Artificial Intelligence
BC	Bronchoscopy
CD	Cluster of Differentiation (e.g., CD20, CD117, CD3)
CT	Computed Tomography
EBUS	Endobronchial Ultrasound
ECLS	Extracorporeal Life Support
ECMO	Extracorporeal Membrane Oxygenation
IHC	Immunohistochemistry/Immunohistochemical
MRI	Magnetic Resonance Imaging
MTS	Malignant Tracheal Stenosis
NBI	Narrow Band Imaging
PAX8	Paired Box Gene 8
PET-CT	Positron Emission Tomography–Computed Tomography
R0	Complete Surgical Resection (Microscopically Negative Margins)
R1	Microscopic Incomplete Surgical Resection (Microscopically Positive Margins)
R2	Macroscopic Incomplete Surgical Resection (Gross Residual Tumor)
SCC	Squamous Cell Carcinoma
TNM	Tumor, Node, Metastasis (Staging System)
TTF-1	Thyroid Transcription Factor-1
VATS	Video-Assisted Thoracoscopic Surgery
